# Nurses’ understanding of quality documentation: A qualitative study in a Mental Health Institution

**DOI:** 10.4102/curationis.v48i1.2737

**Published:** 2025-05-22

**Authors:** Nkhensani F. Mabunda, Itumeleng G. Masondo, Andile G. Mokoena-de Beer

**Affiliations:** 1 Department of Nursing, Faculty of Health Sciences, Sefako Makgatho Health Science University, Pretoria, South Africa

**Keywords:** clinical information, medical record, nursing documents, quality nursing records

## Abstract

**Background:**

Nursing documentation is an integral part of nursing practice that is planned and delivered to individual patients by qualified nurses to provide evidence of the standard of care. The quality of nursing documentation is the inscriptions of all categories of nurses, including students, to record nursing care to facilitate continuity of care and patients’ safety.

**Objectives:**

This study aimed to explore and describe the psychiatric nurses’ comprehension of the quality of nursing documentation in the selected mental health institution in Gauteng province.

**Method:**

The qualitative, explorative-descriptive and contextual design was used. The target population was all nurses directly involved in patient care. Individual face-to-face semistructured interviews were used to collect data. Braun and Clarke’s (2022) six steps of the thematic descriptive analysis method were adopted to allow the second author to identify themes and recapitulate data.

**Results:**

The two themes and subthemes that emerged from the findings include nurses’ understanding of the impact of quality documentation on patient care outcomes and support needs to improve the quality of nursing documentation.

**Conclusion:**

Understanding the quality of nursing documentation is an essential element for producing continuous clinical communication and reflection on the everyday activities of nursing care that are planned and implemented on individual patients’ progress reports.

**Contribution:**

The study contributes to nursing practice, as its results can be used to measure the quality of the primary source of clinical information improvements, allowing healthcare professionals to communicate with each other about a patient’s care.

## Introduction

Nursing documentation is a daily activity in nursing care that is planned and implemented as a key source to record patients’ information to meet professional accountability (Suroso & Santosa 2023:2). Although documentation is an integral part of the nursing practice, it is commonly implemented at the time of each patient interaction to provide evidence of the standard of care (Health Professions Council of South Africa 2022:1). According to Moldskred, Snibsøer and Espehaug (2021:2), the quality of nursing documentation can be reviewed as the inscriptions of all different categories of nurses, including students, to record nursing care during admission, treatment and discharge in order to increase better continuity of patient care. The importance of documenting nursing care is stipulated in the South African Nursing Council (SANC) Regulation R387, emphasising that nurses are responsible for their acts and omissions to keep timely, clear, concise, legible and accurate records of all nursing actions rendered to the patients. Failure to comply constitutes professional misconduct and leads to disciplinary actions (SANC 2005:8).

Several studies have been conducted regarding nursing documentation in the health context. A quantitative study analysed the relationship between nursing documentation and nurses’ work motivation in the coastal areas of Berau Regency, indicating no significant relationship between nurses’ work motivation and the implementation of nursing documentation (Ningrum 2024:50). This contrasts with Bolado et al.’s (2023:10) study to assess the documentation practice and their associated factors. The study found that work experience and having in-service training had significant associations with documentation practice. According to Papinaho, Häggman-Laitila and Kangasniemi (2022:140), it constitutes unprofessional nursing conduct when nurses fail to meet the required standards of nursing documentation, as this affects the quality of patient care.

A study by Maghsoud et al. (2022:6) highlighted nurses’ workload as a factor that affects the quality of nursing documentation. The same study identified that regular supervision and provision of strategies, such as in-service training, can improve the quality of nursing care. This correlates with Oliveira and Peres’s (2021:3) study, which found that staff in-service training and deployment of a new approach can improve the quality of nursing documentation. Additionally, another study found that knowledge, a lack of motivation and training, the convenience of documentation tools, and the use of electronic systems are significant factors associated with documentation quality (Demsash et al. 2023:6).

Correspondingly, a study was conducted by Michl, Paterson and Bail (2023:3451) to understand nurses’ feelings about a documentation audit and how it relates to their professional role. The study identified that the audit experience sorely tests nurses’ psychological well-being, which leads to burnout, stress and demotivation (Michl et al. 2023:3451). The same study found that auditing nursing documentation is the best way to identify the gaps without threatening the professional role of nurses. Similarly, Wakefield et al.’s (2021:66) study identified that feedback from documentation audits among nurses can harm their psychological well-being. This warrants further explanation to understand the impact of quality nursing documentation on continuous patient care outcomes.

An integrated review was conducted by Tadzong-Awasum and Dufashwenayesu (2021:5) to identify the issues related to the nursing documentation in sub-Saharan African countries and found that the nursing process tool was not adequately implemented. The same study also identified several factors, such as inadequate staffing, a lack of knowledge and stressful working environments that affect the quality of nursing documentation in most sub-Saharan countries in Africa. It was recommended that nursing managers find ways to capacitate nurses to acquire professional knowledge to improve the quality of nursing documentation (Tadzong-Awasum & Dufashwenayesu 2021:5). Additionally, a systematic review study was conducted to identify factors associated with nursing documentation in sub-Saharan countries. Age, gender, work experience, education level and working hours per week were identified to be factors associated with inadequate nursing documentation that were reported as threatening the quality of nursing care and patient safety (Imam et al. 2023:21).

Various studies have been conducted in South Africa regarding the quality of nursing documentation, which is a significant aspect of promoting continuous quality nursing care. A qualitative, exploratory-descriptive study by Nyelisani, Makhado and Luhalima (2023:6), to explore and describe the nurses’ understanding of quality care in the selected hospitals of Limpopo province, identified a lack of resources and staff shortage as major factors that affect the quality of documentation. Therefore, the same study recommended that nurses need to be trained in holistic care skills and evidence of care provided to patients based on a joint understanding of the patient’s physical, psychological and spiritual aspects in the nursing records (Nyelisani et al. 2023:6). The quantitative, descriptive study conducted in KwaZulu-Natal to describe the accuracy of patients’ file recordings found that age and lack of knowledge, years of experience and education level were identified as the factors associated with inadequate nursing documentation that can put patients at risk and affect quality care and further influence the process of recording, reviewing and reporting patients’ recovery process (van Graan, Scrooby & Bruin 2020:5).

### Purpose of the study

The study aimed to explore and describe psychiatric nurses’ comprehension of quality nursing documentation in a selected mental health institution in Gauteng province.

## Research methods and design

A qualitative explorative-descriptive and contextual design was relevant for the researchers to gain insight into the psychiatric nurses’ comprehension of the quality of nursing documentation (Polit & Beck 2021:463). The design enabled an in-depth understanding of documentation quality in its unique context (Doyle et al. 2020:450).

### Setting

The study was conducted in a selected mental health institution in Pretoria West, Gauteng. The institution has 24 units with a 32-bed capacity for 778 mental healthcare users (MHCUs), admitting acute and continuous care adults, adolescents, children, forensic and observation MHCUs. The institution also serves as a referral facility from Mpumalanga, Limpopo and North West provinces. The institution had 847 nurses with 37 operational managers, 350 professional nurses, 130 enrolled nurses and 330 enrolled nursing auxiliaries. A unit with 32 MHCUs usually has one operational manager, three professional nurses, two enrolled nurses and four enrolled nursing auxiliaries on a day shift.

### Study population and sampling strategy

The population were all nurses. The target population consisted of all nursing categories, both males and females of all ages, providing mental healthcare to MHCUs. The inclusion criteria were all nurses directly involved in patient care, on day duty, and all were willing to participate in the study voluntarily. All nurses who were not directly involved with in-patient care, who were not on duty, or willing to participate in the study were excluded. A nonprobability purposive sampling technique was used to select the participants who were most likely to yield valuable information based on their knowledge and experience in the area of interest (Campbell et al. 2020:654). The second author approached the nurses through unit managers to explain that this study has 13 participants who were interested in the research topic and who corresponded with the author to arrange their appointments.

### Data collection

The second author conducted individual face-to-face semistructured interviews from August to November 2023 to explore the ideas and thoughts of the participants. One open-ended question, ‘What is your understanding of quality nursing documentation’ was posed, followed by probing questions to ascertain additional information and clarification (Ruslin et al. 2022:24). The interviews were conducted in English and lasted between 35 min and 45 min. They were held in a private room within the institution within a scheduled time for appointment. A pilot interview with three nurses was conducted to test the efficacy and relevance of the designed and probing questions before the main study commenced. No changes in the interview process were made during the actual interview because no discrepancies were experienced during the pilot test. Data obtained during the pilot interviews were analysed and included in the study because the initial study sample was too small (Shakir & Rahman 2022:221). A voice recorder was used to secure the information with the participants’ consent. Data adequacy was reached by participant number 11, but two additional participants were interviewed to confirm data saturation (Daher 2023:3). The second author prepared the raw data and transcribed the recorded interviews verbatim to replicate their exact words (Fearnley 2022:3). Field notes were used to record observations of the participants’ reflective and nonverbal communication based on the second authors’ experiences throughout the interviews (Busetto, Wick & Gumbinger 2020:3).

### Data analysis

In this study, Braun and Clarke’s (2022:65) six steps of thematic descriptive analysis were adopted to allow the second author to identify themes and recapitulate data (Lester, Cho & Lochmiller 2020:97). In the first step, the second author listened to audio recordings and immersed himself in the data to familiarise himself with the data. Initial codes were generated, expressed by substantial notes in labels for essential data features relating to the broad research question. Themes were created by identifying similarities in the data and collating the codes. Themes were reviewed by checking coded extracts and the complete data sets to discard and collapse some themes into one theme. The essence of each theme was identified, and concise information was refined to define and name the theme. Finally, the report was produced by weaving together analytic narratives and data extracts to write a coherent and persuasive account of the research. An independent coder was consulted to analyse the data and he met with the second author to discuss the analysis and to reach consensus about the themes and to eliminate bias in the results. The results were presented with quotations from participants and discussed with references to the existing literature.

### Ethical considerations

Authors were granted permission to conduct the study from the Sefako Makgatho University Research Ethics Committee reference no SMUREC/H/241/2023; Gauteng province Department of Health, as well as from the Head of the Institution of the selected MHI, Ref No: GP 202307091, where the study was conducted. The following ethical principles are adhered to promote research participants’ well-being as a top priority in qualitative research (Mirza, Mirza & Bellalem 2023:244). These include voluntary participation and informed consent, respect for human dignity, the right to privacy and confidentiality, anonymity, beneficence and justice. Participants’ right to self-determination was respected by providing detailed explanations about the research and objectives before signing the consent form, and they had the right to ask questions. They were advised that they could withdraw at any time from the study if not comfortable, even if they had already signed the consent form to treat them equally. Those who declined participation were treated nonjudgmentally.

Participants’ privacy was maintained by discouraging participants from sharing information with anyone and from reporting in a manner that identified participants. To adhere to participants’ rights to full disclosure and confidentiality, each participant was assigned a code, such as P1 for participant number one. The author did not ask questions that could affect participants psychologically regarding their personal views, fears, or weaknesses. In this study, participants benefited indirectly as they helped to gain insights that may increase the knowledge and understanding, which may enhance and optimise the quality of nursing documentation in nursing practice (Bitter et al. 2020:127). Data were stored securely, with encrypted passwords in the Nursing Department’s personal computers at multiple locations. The data were backed up for 5 years, and it will be automatically deleted.

## Results

The results presented in the demographic profile were first followed by the findings of the study. The demographic profile of the interviewed participants is shown in Table 1.

**TABLE 1:** Demographic profile of the participants.

Participant	Age (years)	Gender	Years in the profession	Nursing category
P1	55	Female	28	Operational manager
P2	32	Male	09	Professional nurse
P3	29	Female	06	Professional nurse
P4	39	Female	10	Operational manager
P5	36	Male	07	Enrolled nursing auxiliary
P6	50	Female	26	Professional nurse
P7	36	Female	08	Enrolled nursing auxiliary
P8	34	Male	12	Enrolled nursing auxiliary
P9	32	Female	08	Enrolled nurse
P10	38	Female	06	Enrolled nurse
P11	32	Female	08	Professional nurse
P12	32	Female	04	Professional nurse
P13	32	Male	09	Professional nurse

Participants’ age ranged from 29 to 55 years; the majority (nine) were females and four were males; their years in the profession ranged from 4 to 28. Of the 13 participants, two were operational managers, six were professional nurses, two were enrolled nurses and three were enrolled nursing auxiliaries.

Table 2 shows the two themes and subthemes that emerged from the study’s findings. Additionally, each subtheme is substantiated by direct quotations from the participants.

**TABLE 2:** Themes and subthemes.

Themes	Subthemes
1. Nurses’ knowledge of the quality documentation on patient care outcomes	1.1 Insight of the quality of nursing documentation 1.2 Benefits of quality nursing documentation 1.3 Consequences of poor-quality nursing documentation
2. Support needs to improve the quality of nursing documentation	2.1 Innovative and efficient quality improvement initiatives 2.2 Strengthened supervision

### Themes and subthemes that emerged from the results

#### Theme 1: Nurses knowledge of the quality documentation on patient care outcomes

The participants had varied opinions regarding their knowledge of the quality documentation on patient care outcomes. This finding is confirmed by the following insight of the quality of nursing documentation, benefits of quality nursing documentation and consequences of poor-quality nursing documentation.

**Subtheme 1.1: Insight of the quality of nursing documentation:** The participants shared their understanding of quality nursing documentation, which encompasses evidence of all the activities performed on the patient. They expressed that quality nursing documentation is a continuous communication that requires all nurses to participate in nursing practice:

‘The nursing documentation, uh, it’s a tool that we use in order to improve our nursing process. You write what you saw. Okay. Uh, what you understand about user, about the condition of the patient.’ (P8, 34 years old, male)

‘Nursing documentation is communicating with other nurses that are not here. We record everything that we have done from the morning routine including vital signs meals served and medication to communicate the patient’s progress in the nursing documentation.’ (P10, 38 years old, female)

‘Quality nursing documentation, according to my understanding, is that, uh, our documentation as nurses must have a quality which the records, must be able for the next party to be able to see them, whereby they must reflect date, they must reflect time, and they must be kept safe.’ (P9, 32 years old, female)

**Subtheme 1.2: Benefits of quality nursing documentation:** They also indicated that recording everything positively benefits both the healthcare providers and the service users. The participants also indicated they benefit significantly to the extent that the next nurse who will read the document and will understand precisely what has been done to the patient and what to do:

‘Quality nursing documentation for me is when someone has documented something, the next person is able to understand fully what is happening or what has been documented, like it’s done in totality. Let me use the word comprehensive. Comprehensive. It should be able to cover all questions that the next person may have regarding what has been documented.’ (P6, 50 years old, female)

‘Quality nursing documentation, in my perception, would be the records we need to keep or the records we keep in order to improve on the service delivery that we’re giving to the community or in order to improve full future nursing aspirations.’ (P5, 36 years old male)

‘A decent quality record, it’s what. When you are reading it after the nurses have written it, that’s what will give a clear picture of what has happened to the patient and what was that to solve the problem.’ (P4, 39 years old, female)

**Subtheme 1.3: Consequences of poor-quality nursing documentation:** Participants raised their concerns regarding the consequences of poor-quality nursing documentation and its impact on patient care. They reflected that inadequate records delay patients’ progress because the next nurse does not have enough information about the patient to direct what should be done:

‘The impact is negative, as you can note from what I’ve mentioned before the continuity of care can be disrupted by this. Even worse sometimes, [*t*]he negative impact can change the direction of care because it can communicate the wrong message.’ (P2, 32 years old, male)

‘Whatever it is not written, it was not done for the patient. If the case arises or an incident happens, obviously will have to rely on the recording for the day.’ (P3, 29 years, female)

‘… [*H*]ence it causes prolonged stay to the patient because with our documentation there is no proper communication amongst all the multidisciplinary team members. You don’t know when the next staff come, they don’t get information about the patient …’ (P1, 55 years old, female)

### Theme 2: Support needs to improve the quality of nursing documentation

The support needed to improve the quality of nursing documentation was divided into two subthemes namely, innovative and efficient quality improvement initiatives and strengthened supervision.

**Subtheme 2.1: Innovative and efficient quality improvement initiatives:** Participants suggested some innovative and efficient quality improvement initiatives to identify the gaps linked to inappropriate documentation. Staff satisfaction and in-service training were reflected as innovative and efficient quality improvement initiatives:

‘Staff satisfaction should be conducted as it plays a role in motivating staff. If I’m not motivated to come to work or I’m not motivated to do something it means there is a problem. It will be easier to identify what’s wrong and then fix the problem. Maybe it can be fixed. Maybe people will be motivated to do the right thing.’ (P11, 32 years old, female)

‘We can go to the in-service at least once a month, through that, it helps a lot. It helped a lot for us to understand more about the progress report writing.’ (P9, 32 years old, female)

They reflected that auditing themselves assists them in understanding the need to improve to the extent that the progress report must reflect everything about the patient:

‘… [*I*]t should be continuous. For example, if we do the audits on weekends, then we always identify the same problem, a nursing problem not recorded in Cardex.’ (P6, 50 years old, female)

‘For us to achieve quality documentation or quality nursing, it would start off by … a course in emotional intelligence … when they have mastered the emotional intelligence then that would come to, that people would have a boosted morale.’ (P5, 36 years old, male)

**Subtheme 2.2: Strengthened supervision:** Participants expressed a need for strengthened supervision as a significant aspect of assisting managers in consistently checking how documentation is done in the unit. Participants reflected that observing all patients’ records and the way they give feedback is important in assisting managers in planning the in-service training, focusing on what they have observed:

‘Maybe they should do the rounds in order to check the documentation as to how things are being done in the unit, If it is not correct, then intervene on the problem.’ (P3, 29 years. Female)

‘That also is dependent on the supervisors, or the one above, if they always talk about the negative and not indicate the positive. That would also affect the morale of the people.’ (P5, 26 years, male)

‘Close supervision will give us the opportunity to comply with recording when the supervisors plan on what they have observed. And by doing the right thing, it will benefit the patient ongoing treatment.’ (P 13, 32 years, male)

## Discussion

The authors gained insight into professional psychiatric nurses’ comprehension of the quality of nursing documentation. The first section in the results presents the demographic description of participants. This allows readers to determine to whom research findings are generalised to and allows them to compare across replications of this study and add to the body of knowledge and understanding of universal propositions and discrepancies that exist among populations (Chow et al. 2023:419; Pateman, Dyke & West 2021:8). This study revealed that most participants were female professional nurses aged 29 to 39 years with 4 to 9 years of experience in the nursing profession. The gender disproportion has long been observed, in a predominantly female-dominated nursing profession (Masibo, Kibusi & Masika 2024:1).

The second section explores the psychiatric nurses’ comprehension of the quality of nursing documentation in the selected mental health institution in Gauteng province. As noted in the literature, the quality of nursing documentation was emphasised in the principles for nursing documentation for patient safety, communication and legal accountability. In this regard, nurses are obliged to ensure that patients’ records are clear and accurate to support clinical decision-making and for communication with the healthcare team (American Nurses Association 2010:7). The study’s findings revealed that nurses understand the impact of quality documentation on patient care outcomes. Several studies were conducted concerning the quality of nursing documentation. Bjerkan, Valderaune and Olsen’s (2021:1) study tries to understand barriers to patient safety in nursing documentation and identified that factors such as individual, technological organisational and social barriers, negatively influence effective documentation practices and information exchange. This may impact the quality of patient care outcomes. Participants indicated that the quality of nursing documentation plays an important role in continuous patient care, providing a clear picture of what has happened to the patient and what should be done.

However, the quality of nursing documentation remains a relevant topic for research. Research shows that audit and personal feedback were highlighted as a strategy to improve the quality of nursing documentation to achieve successful documentation (Bjerkan et al. 2021:1; Bunting & De Klerk 2022:7; Perez et al. 2022:280). In addition, it is believed that the quality of nursing documentation plays a critical role in promoting formal, reliable and effective communication between health professionals; hence it also serves as the indicator of the service and facilitates continuity of patient care and patient safety (Abd El Rahman, Ibrahim & Diab 2021:2).

Participants demonstrated positive insight into the quality of nursing documentation. They also indicated that complying with the principles of record keeping entails being able to record the information that is comprehensive and concise as a vehicle of communication within the interprofessional healthcare team to guide them to continue caring for the patient (Lapum et al. 2020:8). This study revealed that their understanding of quality nursing documentation has a positive impact that benefits both the healthcare providers and the service users, thus enabling them to identify the unforeseen risk of mismanaging the MHCUs. Such risk can lead to undesirable outcomes, including impending legal claims, and the defences of both patients and healthcare providers (Nkechi 2021:7).

Research suggests that nursing documentation has repeated gaps or inconsistencies that are difficult to locate and interpret, thus contributing to patient safety errors. This study highlighted some consequences of poor-quality nursing documentation as a significant aspect of quality patient care. Participants indicated that they audit themselves to identify the gaps linked to inappropriate documentation. Literature shows that inappropriate documentation affects quality, continuity and patient safety in the nursing practice, which is against the acts, omissions and commission (Bail et al. 2020:37). The same authors recommended that nurses should be trained to document patient records effectively to promote patient safety and the quality of care using electronic nursing documentation to reduce documentation error rates, incidents and time-savings. To achieve a good result of optimal nursing documentation, this study recommends regular in-service training to capacitate nurses to prevent poor documentation.

The study’s findings highlighted the suggestion to improve the quality of nursing documentation. Participants indicated that support needs are a significant aspect that plays a key role in ensuring that all patient records are accurate. In addition, internal record auditing and feedback were considered to be a critical aspect of motivating nurses to attend in-service training to support them in improving the quality of nursing documentation. It is believed that in-service training and workshops increase the knowledge and overall understanding of the contents within the nursing practice (Aini et al. 2023:125; Javanbakht et al. 2025:4). A study by Birhanu et al. (2024:13) to assess the practice of documentation and its associated factors revealed that the self-reported quality improvement checklist enabled them to collectively identify and explore barriers regarding documentation practice. The same study highlighted that in-service training on documentation standards for all nurses, motivated them to enhance their positive attitudes and increase their knowledge to develop a culture of respectable nursing documentation (Birhanu et al. 2024:13).

A staff satisfaction survey specifically for nursing documentation was suggested as a critical aspect of innovative and efficient quality improvement initiatives to identify gaps in how nurses can be supported to improve the quality of nursing documentation. Research shows that participation in the staff satisfaction survey is a direct feedback tool that allows the employees to share their opinions and experiences and make suggestions (Ashley & Brijball Parumasur 2024:68). Hence, it is important to assist managers in understanding the satisfaction level of employees to improve the quality of satisfaction and how staff can be motivated to partake in ongoing in-service education and professional development programmes that are scheduled within the institution (Rony 2024:1).

In addition, this study identified strengthened supervision to observe all patients’ records as a significant aspect to assist managers in planning in-service training and focused on nursing documentation. These findings are similar to those of Yulianita et al. (2020:7581), who revealed that ongoing observation and giving feedback are active supervision activities that motivate and allow nurses to discuss how they can develop consistent patient records. Another study supported this by highlighting the importance of supervisory support as a significant aspect in facilitating the implementation of relevant policies and protocols to reduce nurse burnout while improving the quality of nursing practice (Kim & Lee 2023:1).

### Strengths and limitations

The inclusion of all nursing categories in the research sample was significant in unravelling the knowledge among nurses. This study was qualitative and contextual. Therefore, the use of one research method limited the analysis because triangulation could yield rich findings generalisable to other institutions that did not form part of the sample of this study.

### Recommendations

The following recommendations were made to enhance the quality of nursing documentation in mental health institutions. Mental health institutions should collaborate and establish a committee to develop and oversee standardised documentation formats and policies across all departments. They should develop and implement regular, mandatory training sessions on documentation skills for all nursing categories to improve practice quality. In addition, large-scale studies should be conducted across multiple mental health institutions to enhance the generalisability of the findings.

### Implications for practice

Findings from this study may guide multiple mental health institutions across different provinces to enhance generalisability and identify potential provincial variations in documentation practices. Comparative studies investigating effective documentation practices in mental health contexts versus general healthcare settings could highlight unique challenges and needs in documentation.

## Conclusion

Complete, correct and timely documentation is essential for continuous clinical communication and reflection on the everyday activities of nursing care that are planned and implemented on individual patients’ progress reports. There is a need to put effort towards improving the quality of nursing documentation by providing in-service training on documentation standards and motivating nurses regarding documentation to prevent unforeseen risks of mismanagement and legal claims.
